# A Commentary on Narayan and Mallinson’s (2022) “Transcultural Nurse Views on Culture-Sensitive/Patient-Centered Assessment and Care Planning: A Descriptive Study”

**DOI:** 10.1177/10436596221103248

**Published:** 2022-06-22

**Authors:** Helga Martins, Revital Shapira, Sílvia Caldeira

**Affiliations:** 1 Instituto Politécnico de Beja, Universidade Católica Portuguesa, Lisboa, Portugal; 2 Hebrew University School of Nursing, Jerusalem, Israel; 3 Universidade Católica Portuguesa, Lisboa, Portugal

Recently, an article was published in *Journal of Transcultural Nursing* by Narayan and Mallinson (2022) entitled “Transcultural Nurse Views on Culture-Sensitive/Patient-Centered Assessment and Care Planning: A Descriptive Study.” This is an outbreaking qualitative study using a nursing focus group approach which was conducted at the Transcultural Nursing Society Conference. The pioneering vision underlines with the perspective that combines culture-sensitive (CS) and patient-centered care (PCC) regarding assessment and the planning of care in culturally diverse settings.

This article addresses a need in an outgoing reality that health contexts are increasingly culturally diverse. However, this situation raises up new challenges that require respond to this diversity, based on an adequate reply by nurses in clinical practice. The findings of this study provide us with relevant information. The major contribution of this study relates to the variety of attitudes (caring and humility), knowledge (self-knowledge, cultural norms/individuality and cultural assessment and care planning), and skills (relationship-building skills, assessment skills and care planning skills) concerning CS/PCC that are necessary for nurses in clinical practice (Narayan & Mallinson, 2022). It was shown in different studies that even when nurses were familiar with and sensitive to the impact that cultural differences may have on a patient’s health care priorities and practices, they often did not know how to incorporate cultural competence principles into their practice. These underline the complexity of competences that nurses must have in culturally diverse contexts, so it is necessary to acquire (or to have) a set of attitudes, knowledge, and skills that allow a deep understanding of human responses to life processes and health problems, in pursuance of enhancing an effective health outcome. This wide range of competences must be established in the curriculum of nurses by nurse educators. In this regard, Sommers and Bonnel (2020) stated that nursing educators need to be ready for a culture-sensitive care and embrace personalized and active methods and teaching strategies, keeping self-awareness of the resources that are available within cultural diversity. In addition, nurse leaders should make a step forward and promote a CS and PCC environment in clinical practice, which has a huge impact in quality of care (Richard-Eaglin, 2021; Wang & Dewing, 2021). These two previous arguments are fundamental to ensure a future that leverages the CS and PCC dyad in a culturally diverse setting.

Another interesting point of view by Narayan and Mallinson (2022) is that caring plays a vital role to CS/PC assessment and care planning. Karlsson and Pennbrant (2020) also stated that caring is the core of nursing and is a specific attribute of nursing.

In this article, Narayan and Mallinson (2022) focused their attention to the nursing process, particularly in assessment and planning in culturally diverse settings. Less attention seems to have been given to nursing diagnosis. Still, it is also important to have nursing diagnosis culturally accurate. Nurses should be aware of patients’ human responses in a culturally diverse setting. Therefore, nursing diagnosis validation studies in different cultural backgrounds are critical in promoting accuracy and planning cultural sensitive interventions. Miguel et al. (2019) reinforce the need for the development of accurate nursing diagnosis and to perform a more completed examination with the use of more axial terms.

In view of this scenario, it has become essential to go further and step by step in building foundations for the implementation of CS and PCC to respond as promptly according to the human responses of patients to health/disease process. Only then will we have the ability to improve the quality of nursing care in patients since we are living in societies with a progressive increase in cultural diversity. The study of Narayan and Mallinson (2022) was limited by its small size sample, and this subject should be further investigated in larger communities all over the world.
